# Antioxidant and Antibacterial Activities of Crude Extracts and Essential Oils of *Syzygium cumini* Leaves

**DOI:** 10.1371/journal.pone.0060269

**Published:** 2013-04-12

**Authors:** Amal A. Mohamed, Sami I. Ali, Farouk K. El-Baz

**Affiliations:** Plant Biochemistry Department, National Research Centre (NRC), Dokki, Cairo, Egypt; University of Sydney, Australia

## Abstract

This research highlights the chemical composition, antioxidant and antibacterial activities of essential oils and various crude extracts (using methanol and methylene chloride) from *Syzygium cumini* leaves. Essential oils were analyzed by gas chromatography-mass spectrometry (GC-MS).The abundant constituents of the oils were: α-pinene (32.32%), β-pinene (12.44%), trans-caryophyllene (11.19%), 1, 3, 6-octatriene (8.41%), delta-3-carene (5.55%), α-caryophyllene (4.36%), and α-limonene (3.42%).The antioxidant activities of all extracts were examined using two complementary methods, namely diphenylpicrylhydrazyl (DPPH) and ferric reducing power (FRAP). In both methods, the methanol extract exhibited a higher activity than methylene chloride and essential oil extracts. A higher content of both total phenolics and flavonoids were found in the methanolic extract compared with other extracts. Furthermore, the methanol extract had higher antibacterial activity compared to methylene chloride and the essential oil extracts. Due to their antioxidant and antibacterial properties, the leaf extracts from *S. cumini* may be used as natural preservative ingredients in food and/or pharmaceutical industries.

## Introduction

In a biological system, an antioxidant is defined as any substance that when present at low concentrations compared with those of an oxidizable substrate, significantly delays or prevents oxidation of that substrate. Recently, increasing attention has been focused on the use of natural antioxidants, such as ascorbic acid, tocopherols, phenolic compounds including flavonoids**,** phenolic acids, and volatile compounds for preventing oxidation of biomolecules which can lead to cell injury and death [1]. The medicinal properties of some plants have been investigated throughout the world**,** due to their potent antioxidant activities. Reactive oxygen species (ROS) including singlet oxygen (^1^O_2_), superoxide ion (O_2_^−^), hydroxyl ion (OH ), and hydrogen peroxide (H_2_O_2_) are highly reactive and toxic molecules generated in cells under normal metabolic activities. ROS can cause oxidative damage to proteins, lipids, enzymes, and DNA molecules [2]. Living cells possess powerful scavenging mechanisms to avoid excess ROS-induced cellular injury, but with ageing and under influence of external stresses, these mechanisms become inefficient, and dietary supplementation by antioxidants is required [3].

In recent years, due to toxicological concerns associated with the use of synthetic substances in food and increasing awareness about natural foods, there has been an increased interest in the use of natural substances as food preservatives and antioxidants [4]. Flavonoids, tannins, anthocyanins and other phenolic constituents present in food of plant origin are potential antioxidants [5], [6]. Food rich in antioxidants plays an essential role in the prevention of some cancers and cardiovascular and Alzheimer's diseases [7]. Therefore, the search for antioxidants, hypoglycemic and anticancer agents in vegetables, fruits, tea, spices and medicinal herbs have attracted increasing attention. In this context, many aromatic plants, and particularly their essential oils, are being evaluated for antioxidant activity. *Syzygium cumini* Linn (family Myrtaceae), commonly known as “ Jamun ” has promising therapeutic value with various phytoconstituents such as tannins, alkaloids, steroids, flavonoids, terpenoids, fatty acids, and vitamins. The seeds of *S. cumini* have been reported to be rich in flavonoids, which account for the scavenging of free radicals and a protective effect on antioxidant enzymes [8], and they have also been found to have high total phenolic content with significant antioxidant activity [9], [10].

The chemical composition and antioxidant activity of *S. cumini* fruits have been also studied recently and tannins extracted from *S. cumini* fruit showed a very good DPPH radical scavenging activity and ferric reducing/antioxidant property [11]. According to nutritionists, the fruit of *S. cumini* is rich in carbohydrates, minerals and vitamins, with glucose and fructose as principal sugars. The fruit is also rich in minerals such as manganese, zinc, iron, calcium, sodium and potassium.

The ripe *S. cumini* fruit has many therapeutic properties such as liver stimulation, digestive, carminative, coolant and hypoglycemic effects [12]. Its leaves contain essential oils with a pleasant odour. The oil contains terpenes, dipentenes, sesquiterpenes, ellagic acid, isoquercitin, quercetin, kaempferol and myricetin in different concentrations [13]. The barks, leaves and seeds extracts of *S. cumini* have been reported to possess anti-inflammatory [14], and antidiarraheal effects [15]. The leaves have been extensively used to treat diabetes and constipation [16], fever, gastropathy, strangury and dermopathy [17].

Active ingredients of plants against microorganisms are frequently secondary metabolites (i.e. alkaloids, glycosides, etc.) that are present in abundance in herbs and spices [18]. *Syzygium* species have been reported to possess antibacterial activity [19]. Aqueous and methanol extracts of *Syzygium* species have been shown to inhibit the growth of some fungal microorganism implicated in skin diseases, such as *Candida albicans*, and *Trichophyton rubrum* [20]. Therefore, the objectives of the present study were (i) to determine the chemical composition of essential oils of *S. cumini* leaves; (ii) to determine the contents of total phenolic and flavonoids of essential oil and two organic extracts of *S. cumini* leaves; and (iii) to evaluate the *in vitro* antioxidant and antibacterial properties of the essential oils and the two different organic extracts.

## Materials and Methods

### Chemical reagents and solvents

Folin-Ciocalteu reagent, 2, 2′-diphenyl-1-picrylhydrazyl (DPPH), sodium carbonate, aluminum chloride and methanol were purchased from Sigma Chemical Co., Ltd (St. Louis, MO, USA). Butylated hydroxytoluene (BHT) was purchased from Sigma-Aldrich. All other reagents and solvents were of analytical grade.

### Collection of plant materials

Fresh leaves of *S. cumini* were collected from the farm of Faculty of Agriculture, Cairo University- Egypt. Voucher specimens were deposited in the Herbarium of Phytochemistry and Plant Systematic Dept, National Research Centre – Egypt for *S. cumini* (No, 14562).

### Microbial strains

Six bacterial strains were obtained from the American type culture collection (ATCC; Rockville, MD, USA) as well as the culture collection of the Micro Analytical Centre, Faculty of Science, Cairo University-Egypt. They were *Escherichia coli* ATCC 35218, *Staphylococcus aureus* ATCC 25923, *Pseudomonas aeruginosa* ATCC 29212, *Neisseria gonorrhoeae* ATCC 11778, *Bacillus subtilis* ATCC 12228, *Staphylococcus aureus* ATCC 9341, and *Enterococcus faecalis* ATCC 9763.

All microorganisms were stocked in appropriate conditions and regenerated twice before using.

### Extraction of essential oils from leaves

The leaves of *S. cumini* were air-dried. The dried leaves (100****g) were subjected to hydro-distillation using a Clevenger apparatus for 4****hrs for the isolation of essential oils according to the method recommended by Guenther [21]. The volume of the extracted essential oil was recorded, and two replicate extractions were done. The essential oil yield was 0.56±0.08% (w/v). The extracted essential oils were dehydrated over anhydrous sodium sulfate and stored at 0°C in air-tight glass vials until used for GC-MS analysis.

### Analysis of essential oil compounds

The chemical composition of *S. cumini* leaves essential oils was analyzed using a Hewlett Packard model HP6890 gas chromatograph (Agilent Technologies, Palo Alto, CA, USA) equipped with an HP-5MS (5% phenylpolymethylsiloxane) capillary column (30 m'0.25 mm i.d., film thickness 0.25 μm; Agilent Technologies, USA) interfaced to an HP model 5973 mass-selective detector. The oven temperature was initially held at 50°C and then increased by 2°C/min to 180°C. The injector and detector temperatures were 250 and 280°C, respectively. Purified helium was used as the carrier gas at a flow rate of 1 ml/min. EI mass spectra were collected at 70 eV ionization voltages over the range of m/z 29–300. The electron multiplier voltage was 1150 V. The ion source and quadrupole temperatures were set at 230 and 150°C, respectively. Identification of volatile compounds was performed by comparing the NIST 05 and Wiley 275 libraries data of the peaks with those reported in literature [22]. Percentage composition was computed from GC peak areas on TR-5MS column without applying correction factors.

### Preparation of crude extracts

The air-dried leaves of *S. cumini* were pulverized into powdered form. The dried powder (0.5****g) was extracted by soaking with methanol (Me-OH), and methylene chloride (Me-Cl) separately using orbital shaker for 48****hrs at room temperature. The extracts were filtered through Whatman No.1 filter paper. Residues were re-extracted twice with fresh aliquots of the same solvents. Solvents from the combined extracts were evaporated using a vacuum rotary evaporator [23] and the resulting residues were used for the analyses outlined below.

### Determination of total phenolic content

Total phenolic content in the essential oils and leaf extracts (methanol and methylene chloride) from *S. cumini* was measured using the Folin–Ciocalteu reagent method [24]. Briefly, 200 μl of both crude extracts and essential oils (5 mg oil/ml ethanol) were made up to 3 ml with distilled water then mixed thoroughly with 0.5 ml of Folin- Ciocalteu reagent. After mixing for 3 min, 2 ml of 20% (w/v) sodium carbonate was added and allowed to stand for a further 60 min in the dark. The absorbance of the reaction mixtures was measured at 650****nm, and the results were expressed as mg of gallic acid (GAE)/g of dry weight.

### Determination of total flavonoid content

Total flavonoid content of both crude extracts and essential oils was determined using the aluminium chloride colorimetric method as described by Chang *et al.* [25]. In brief, 50 µl of crude extracts and essential oils (5 mg/ml ethanol) were made up to 1 ml with methanol then mixed with 4 ml of distilled water and subsequently with 0.3 ml of 5% NaNO_2_ solution. After 5 min of incubation, 0.3 ml of 10% AlCl_3_ solution was added and then allowed to stand for 6 min, followed by adding 2 ml of 1 M NaOH solution to the mixture. Then water was added to the mixture to bring the final volume to 10 ml and the mixture was allowed to stand for 15 min. The absorbance was measured at 510****nm.

Total flavonoid content was calculated as quercetin from a calibration curve. The calibration curve was prepared by preparing quercetin solutions at concentrations 12.5 to 100 mg ml^−1^ in methanol. The result was expressed as mg quercetin (QU)/g of dry weight.

### Determination of antioxidant activities

#### a- Ferric reducing power assay

Ferric reducing/antioxidant power (FRAP) was determined following the method reported by Zhao *et al.* [26]. For each extract, (methanol, methylene chloride and essential oils), 100 μl was mixed with 2.5 ml of 200 mM phosphate buffer (pH 6.6) and 2.5 ml of 1% potassium ferricyanide. Mixtures were incubated at 50°C for 20 min, 2.5 ml of 10% trichloroacetic acid was added and the tubes were centrifuged at 10,000 rpm for 10 min. Five millilitres of the upper layer of the solution was mixed with 5.0 ml distilled water and 1 ml of 0.1% ferric chloride. The absorbance of the reaction mixtures was measured at 700****nm. The final results were expressed as μg ascorbic acid equivalent/g of dry weight.

#### b- DPPH radical scavenging assay

Quantitative measurement of radical scavenging properties of *S. cumini* was carried out according to the method of Blois [27]. Briefly, a 0.1 mM solution of 2, 2- diphenyl-1-picryl-hydrazyl (DPPH·) in methanol was prepared and 1 ml of this solution was added to 3 ml of the crude extracts and essential oils at different concentration (10–200 μg/ml). Butylated hydroxytoluene (BHT) was used as a positive control. Discoloration was measured at 517****nm after incubation for 30 min in the dark. Measurements were taken in triplicate. The capacity to scavenge the DPPH· radical was calculated using the following equation:
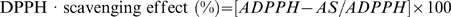
Where, A*DPPH* is the absorbance of the DPPH· solution and A*S* is the absorbance of the solution when the sample extract is added.

The IC_50_ values (concentration of sample required to scavenge 50% of free radicals) were calculated from the regression equation prepared from the different concentrations of both crude extracts and essential oils.

### Assessment of antibacterial activity

A disc diffusion method was used for the antibacterial assay [28]. Sterile nutrient agar plates were prepared for bacterial strains and inoculated by a spread plate method under aseptic conditions. Filter paper discs of 5 mm diameter (Whatman No. 1 filter paper) were prepared and sterilized. 5 µl of the leaf extracts (20 mg/ml), 10 µl of essential oils and 5 µl of Tetracycline (2 mg/ml; used as positive control) were added to each disc. The sterile impregnated discs with plant extracts and essential oils were placed on the agar surface with flamed forceps and gently pressed down to ensure complete contact of the disc with the agar surface. Filter paper discs soaked in solvent were used as negative controls. The antibacterial activity of each extract was expressed in terms of the mean of diameter of zone of inhibition (in mm) produced by the respective extract at the end of incubation period.

### Statistical analysis

Data were expressed as Mean ±SD. A one way variance was used to analyze data, p<0.05 represented significant difference between means (Duncan's multiple range test).

## Results and Discussion

### Chemical composition of the essential oils from *S. cumini* leaves

As shown in the Table 1, 49 compounds, representing about 98.3% of the essential oils were characterized. The oil contained a complex mixture mainly of monoterpene hydrocarbons and oxygen containing mono- and sesquiterpenes. The major compounds that were identified by GC–MS were α-pinene (32.32%), β-pinene (12.44%), trans-caryophyllene (11.19%), 1, 3, 6-octatriene (8.41%), delta-3-carene (5.55%), α-caryophyllene (4.36%), and α-limonene (3.42%), along with some other minor components presented in trace amounts. Volatile oils are very complex mixtures of compounds. The constituents of the oils were mainly monoterpenes and sesquiterpines, which are hydrocarbons with the general formula (C_5_H_8_)_n_. The antioxidant activity of essential oils is of considerable interest as these may preserve foods from the toxic effects of oxidants [29]. Moreover, essential oils have the ability to scavenge free radicals and play an important role in the prevention of some diseases such as brain dysfunction, cancer, heart disease and immune system decline [30]. The high concentration of α-pinene in *S. cumini* leaves oil makes it potentially useful in medicines because this exhibits antibacterial, antifungal, anti-inflammatory, insecticidal and antioxidant properties, and it is used traditionally as flavoring agent and antimicrobial material in food [31], [32]. The present results agree with those of Ayyanar and Subash-Babu [33] who also reported that the essential oils isolated from the freshly collected *S. cumini* leaves contain α-pinene, camphene, β-pinene, myrcene and limonene as a major compounds.

**Table 1:** Composition percent of major volatile oils of *S. cumini* leaves.

No.	Name of constituents	RT	Leaves oil (%)
1	α-Pinene	6.32	32.32
2	Camphene	7.69	0.68
3	β – Pinene	7.96	12.44
4	β – Myrcene	9.06	1.60
5	1-Phellandrene	9.60	0.06
6	(+)-2-Carene	9.68	0.22
7	α-Limonene	9.57	3.42
8	Cinene	9.94	0.79
9	1,3,6-octatriene	10.02	8.41
10	Delta-3-Carene	10.50	5.55
11	β -Ocimene Y	11.18	0.22
12	Trans-beta- Ocimene	11.19	1.32
13	α-Terpinolene	12.19	0.89
14	Alloocimene	13.38	0.78
15	2,6-Dimethyl-2,4,6-octatrienen	13.55	0.43
16	2-Chlorocamphane	14.84	0.09
17	Borneol	15.05	0.16
18	4-Terpineol	15.20	0.25
19	α- Terpineol	15.47	0.25
20	β -Fenchyl Alcohol	15.51	2.12
21	Levo-bronyl-acetate	18.02	0.35
22	Ylangene	19.76	0.07
23	α-Copaene	19.83	0.29
24	Isocaryophillene	20.32	0.04
25	Trans-Caryophyllene	20.36	11.19
26	α – Ylangene	21.23	0.19
27	(+)-Aromadendrene	21.39	0.08
28	(+)-3-Carene,2-acetylmethyl-	21.51	0.12
29	α – Caryophyllene	21.56	4.36
30	α –Muurolene	22.16	0.69
31	Valencene	22.58	0.22
32	Eremophilene	22.62	0.33
33	α –Selinene	22.79	0.32
34	γ-Cadinene	23.07	0.16
35	δ-Cadinene	23.11	0.68
36	Cadina-1,4-Diene	23.57	0.05
37	Aromadendrene	23.79	0.07
38	β -Caryophyllene epoxide	24.22	0.30
39	Caryophyllene oxide	24.57	2.91
40	Globulol	25.17	0.07
41	Camphene	25.24	0.40
42	3-Cyclohexen-1-carboxaldehyde,3,4-dimethyl-	25.48	0.98
43	β –Guaiene	25.86	0.36
44	γ-Gurjunene	26.07	0.21
45	α –Amorphene	26.26	0.57
46	Viridiflorol	26.55	0.59
47	Isoaromadendrene-(V)	26.73	0.36
48	Eudesma-4(14),11-diene	27.03	0.19
49	Pyridine,4-butyl-1-oxide	30.58	0.13
	Total identified compounds	–	98.28

RT: Retention time.

### Total phenolic content

The total phenolic content of *S. cumini* leaves extracted with methanol and methylene chloride and of the essential oils is presented in Table 2. The results indicate that the total phenolic content of various extracts had significant variations, ranging from 6.55 to 14.03 (mg/g d.w). The methanol extract had the highest total phenolic content (14.03 mg/g d.w), while the essential oils extract had the lowest content (1.28 mg/100 mg essential oil). Therefore, these results show that the methanol extract possessed significant activity in releasing most secondary metabolites from leaves. This may be due to the fact that phenolic compounds are often extracted in higher amounts by using polar solvents such as aqueous methanol/ethanol [34]. Differences in the polarity of the extracting solvents could result in a wide variation in the polyphenolic contents of the extract [35]. Methanol is a relatively polar organic solvent compared to methylene chloride, therefore most polyphenolics evaluated in this study are likely to be polar compounds. The key role of phenolic compounds to scavenge free radicals has been emphasized in previous reports [36]. Phenolic antioxidants are products of secondary metabolism in plants, and their antioxidant activity is mainly due to their redox properties and chemical structure, which can play an important role in chelating transitional metals and scavenging free radicals [37] **.** Consequently, the antioxidant activities of plant/herb extracts are often explained by their total phenolic and flavonoid contents.

**Table 2:** Total phenolic, total flavonoids and ferric reducing power contents of methanolic (Me-OH), methylene chloride (Me-Cl) and essential oils (E. oils) extracts of *S. cumini* leaves.

Different extracts	Total phenolic (mg/g d.w)	Total flavonoids (mg/g d.w)	Ferric reducing power (µg/g d.w)
**Me-OH**	14.03±0.55^c^	6.22±0.10^c^	13.14±0.11^c^
**Me-Cl**	6.55±0.32^b^	2.04±0.11^b^	1.22±0.08^b^
**E. oils**	1.28±0.10* ^a^	0.42±0.06** ^a^	0.47±0.05*** ^a^
**LSD 0.05**	0.97	0.1	0.09

* (mg GAE/100 mg essential oil/ml Et-OH).

** (mg QU/100 mg essential oil/ml Et-OH).

*** (µg ascorbic acid/100 mg essential oil/ml Et-OH).

Each value is expressed as mean ± SD. Data with different superscript letters are significantly different (P≤0.05).

### Total flavonoid content

The total flavonoid content of *S. cumini* leaves extracts is given in Table 2. The order of total flavonoids in the different extracts was as follows: methanol extract 6.22 mg/g d.w, methylene chloride extract 2.04 mg/g d.w, and essential oils 0.42 mg/100 mg essential oil. Methanol was more effective for total flavonoid extraction than methylene chloride. It has been recognized that flavonoids show antioxidant activity and that their effects on human nutrition and health are considerable. The mechanisms of action of flavonoids are through scavenging or chelating processes [38]. Compounds such as flavonoids, which contain hydroxyl functional groups, are responsible for the antioxidant effects of plants [39]. The present results are in accordance with the previous report of Zheng *et al.* [40] who stated that the flavonoid content in *Syzygium jambos* extracts was responsible for their antioxidant activity, and a high amount of total flavonoids in the extract suggested that *Syzygium jambos* possesses high antioxidant activity.

### Antioxidant activities

#### a- Ferric reducing power

The presence of a reductant, such as the antioxidant substances in plant extracts, causes the reduction of Fe^3+^ ferricyanide complex to the ferrous form, Fe^2+^. The transformation of iron (III) to iron (II)-reducing activity in the methanol and methylene chloride extracts and the essential oils of *S. cumini* leaves is presented in Table 2. The highest reducing power activity (13.14 μg/g d.w) was found in the methanol extract, followed by the methylene chloride extract (1.22 μg/g d.w) and then the essential oils (0.47 μg/100 mg essential oil). The ferric reducing power of *S. cumini* leaves may be attributed to the phenolic and flavonoid contents of the extracts. In this regards, Banerjee *et al.* [41] stated that, the antioxidant property of the *S. cumini* fruit skin may come in part from the antioxidant vitamins, phenolics, tannins and anthocyanin compounds present in the fruit. These observations confirm the folklore use of *S. cumini* leaves extracts as a natural antibacterial and antioxidant and justify the ethnobotanical approach in the search for novel bioactive compounds. The ability to reduce Fe (III) may be attributed to the hydrogen donation from phenolic compound [42], which is related to the presence of a reducing agent [43]. In addition, the number and position of hydroxyl group of phenolic compounds also govern their antioxidant activity [44].

#### b- Scavenging activity of DPPH radicals

**Figure 1:**
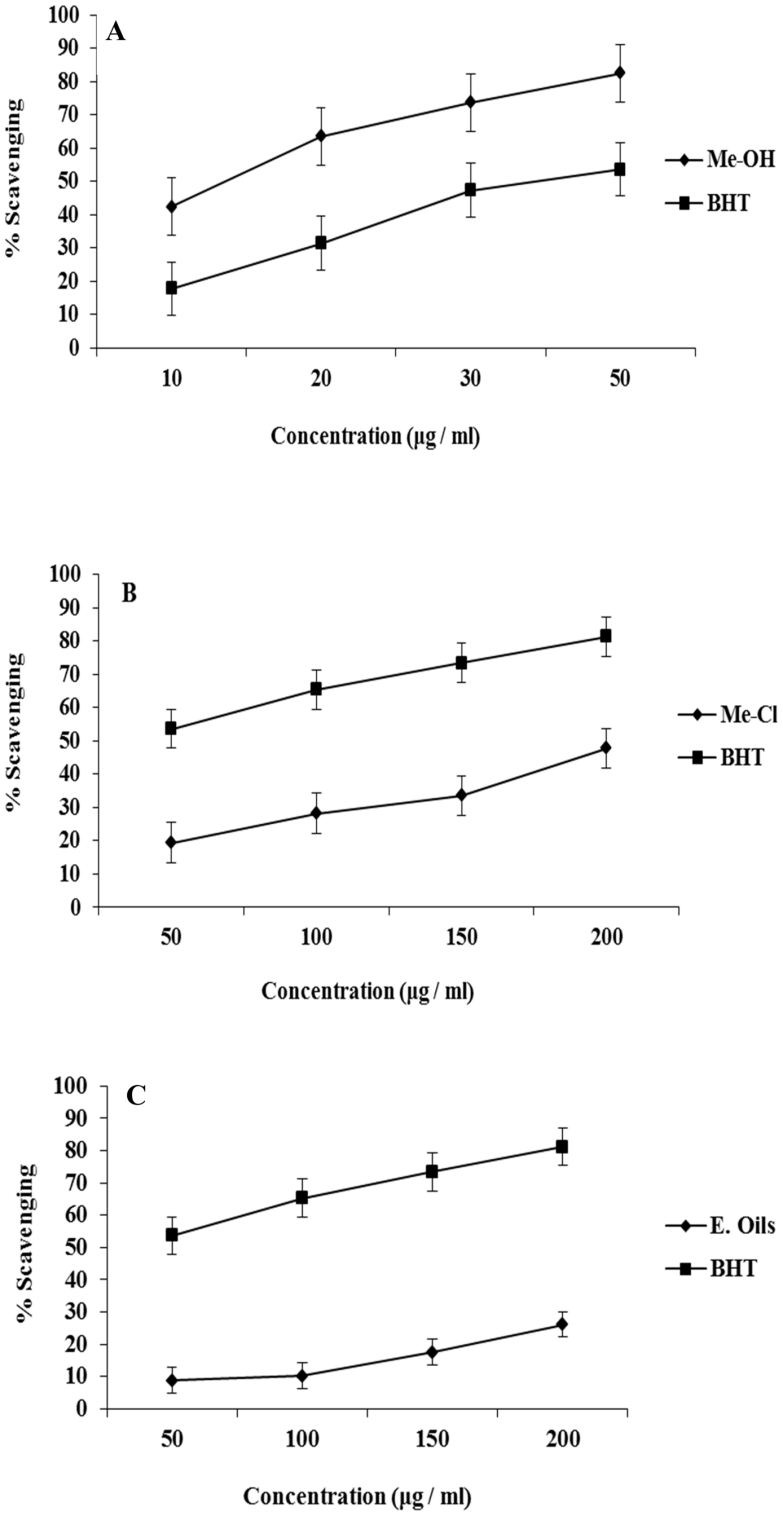
Free radical scavenging activity of extracts from S. cumini leaves: A) methanolic extracts (Me-OH); B) methylene chloride extracts (Me-Cl); C) essential oils (E. oils). Butylated hydroxytoluene (BHT) is included as a positive control. Activity was measure by the scavenging of DPPH radicals. Each value is expressed as the mean ± standard deviation.

DPPH is a stable free radical, which has been widely accepted as a tool for estimating free radical-scavenging activities of antioxidants [45]. The percentages of remaining DPPH in the presence of all extracts at different concentrations are shown in Figure 1. In the present study the methanol extract exhibited higher DPPH scavenging activity (Figure 1A) than the methylene chloride extract (Figure 1B) and essential oils (Figure 1C). The scavenging effects of the methanol and methylene chloride extracts on DPPH radicals increased as extract concentration increased (Figure 1). The reduction in the color of DPPH· radical due to the scavenging ability of the methanol extracts and antioxidant standard (BHT) was significant (P≤0.05). The scavenging effects of different extracts and the standard on the DPPH· radical decreased in the order of methanol extract > BHT > methylene chloride > essential oils, which were 70.45, 67.55, 64.55, and 55.87% at a concentration of 50 μg/ml, respectively. These results indicate that the methanol extracts have a noticeable effect on scavenging free radicals. However, the scavenging effect of BHT was higher than the methylene chloride and essential oil extracts. Phenolic antioxidants are products of secondary metabolism in plants, and the antioxidant activity is mainly due to their redox properties and chemical structure, which can play an important role in chelating transitional metals, inhibiting lipoxygenase and scavenging free radicals [46]. Phenolic compounds are also effective hydrogen donors, which makes them good antioxidants [44]. *Syzygium* species are reported to be very rich in tannins, flavonoids, essential oils, anthocyanins and others phenolic constituents [47], [48]. Fifteen polyphenols and two acylated flavonal glycosides have been isolated from the leaves of *S*. *cuminii* (L) Skeels [49]. The results given in this investigation showed that the phenolic content was higher in polar extracts (methanol) than in less-polar extracts (methylene chloride and essential oils). It seems clear that the presence of polar phenolics is fundamental in the evaluation of free radical-scavenging activity [50].

### Antibacterial activity

Data for the *in vitro* antibacterial properties of methanolic, methylene chloride extracts and essential oils of *S. cumini* leaves are presented in Table 3. The tested extracts and essential oils of *S. cumini* leaves possessed antibacterial activity against both Gram positive and Gram negative bacteria. Among the tested extracts, the methanol extract exhibited the maximum inhibition zones (18–24 mm) against the tested bacteria. Both the methylene chloride extract and the essential oils showed moderate inhibition zones (13–16 and 12–14 mm). The antimicrobial action of the essential oil of *S. cumini* is probably related to the fact that essential oils may disrupt the permeability of cell membranes. Other studies also indicate that these disturb the structure of the cellular membrane and react with the active sites of enzymes or act as a H+ carrier, depleting adenosine triphosphate pool [51].

**Table 3:** Antibacterial activity of methanolic, methylene chloride extracts and essential oils of *S. cumini* leaves.

	Zone of inhibition (mm)			
Bacteria strain	Methanol extract	Methylene chloride extract	Essential oils	Tetracycline antibacterial agent
***Escherichia coli***	22±0.9	13±0.8	12±0.4	30±1.3
***Pseudomonas aeruginosa***	19±0.8	14±0.4	13±0.2	30±0.9
***Neisseria gonorrhoeae***	20±0.7	15±0.8	13±0.4	31±1.4
***Bacillus subtilis***	18±0.5	13±0.4	14±0.2	31±2.0
***Staphylococcus aureus***	24±0.8	15±0.6	12±0.6	32±2.0
*Enterococcus faecalis*	24±1.0	16±0.9	13±0.5	34±1.5

Used concentration: 10.0 µl each of the essential oil or 5.0 µl of 20 mg/ml solution of extracts and Tetracycline. Each value is expressed as mean ± SD.

In this regard, Shafi *et al.* [52] stated that essential oil from the leaves of *S. cumini* showed better antibacterial activity than that from *S. travancoricum,* especially against *Salmonella typhimurium*. In the present investigation the methanol extract was found to be more effective on both Gram positive and Gram negative bacteria, and especially against Gram positive bacteria such as *Staphylococcus aureus* and *Enterococcus faecalis*. Additionally, Shyamala and Vasantha, [53] stated that the *S. cumini* leaf extract showed activity against *Escherichia coli* and *Staphylococcus aureus* and the authors rendered the activity to the presence of tannins and other phenolic constituents in the leaf extract.

## Conclusion

The findings of this study support the view that certain medicinal plants are promising sources of potential antioxidants and may be effective as preventive agents in the pathogenesis of some diseases. The extracts obtained using a high polarity solvent (methanol) were considerably more effective radical scavengers than those using less polarity solvent (methylene chloride), indicating that antioxidant or active compounds of different polarity could be present in leaves of *S. cumini*. There is a need for more research on the use of *S. cumini* essential oils and extracts as preservative agents in different foods. The results presented here should encourage the use of S. cumini leaves for medicinal health, functional food and nutraceuticals applications, due to their antioxidant and antibacterial properties.
